# Mosquito bisection as a variable in estimates of PCR-derived malaria sporozoite rates

**DOI:** 10.1186/1475-2875-11-145

**Published:** 2012-05-02

**Authors:** Desmond H Foley, Genelle Harrison, Jittawadee R Murphy, Megan Dowler, Leopoldo M Rueda, Richard C Wilkerson

**Affiliations:** 1 Entomology Branch, Walter Reed Army Institute of Research, Silver Spring, MD, 20910, USA

**Keywords:** *Plasmodium*, Malaria, Sporozoite rate, Oocyst, PCR, Dissection, *Anopheles*

## Abstract

**Background:**

Highly sensitive polymerase chain reaction (PCR) methods offer an alternative to the light microscopy examination of mosquito salivary glands for the determination of malaria sporozoite rates in wild caught female *Anopheles*. Removal of mosquito abdomens is assumed to eliminate false positives caused by malaria oocyst DNA in the midgut. This assumption has not been tested with current gold standard PCR assays, and for the variety of conditions that specimens could encounter in the laboratory and field.

**Methods:**

Laboratory *Anopheles stephensi* were used that had been infected with *Plasmodium falciparum* 6–7 days and 14 days post infection (p.i.), when oocysts only and oocysts + sporozoites, respectively, are developed. Mosquitoes were killed and immediately frozen, air dried before being frozen, or stored under humid conditions overnight before being frozen, to simulate a range of conditions in the field. Additionally, abdomens were removed anterior to, at, or posterior to the junction of the abdomen and thorax, and both portions were processed using a standard nested PCR of the small sub-unit nuclear ribosomal genes (*ssrDNA*) with products visualized on agarose gels.

**Results:**

Overall, 4.1 % (4/97) of head + thorax samples that were 6–7 days p.i. gave apparent false positives for sporozoites, compared to 9.3 % (9/97) that were positive for abdomens. No positives (0/52) were obtained when similar specimens were bisected anterior to the junction of the thorax and abdomen, compared to 21.2 % (11/52) that were positive for posterior portions. Multiple bands were noted for positives from the ‘Frozen’ treatment and the rate of false negatives due to DNA degradation appears higher under the ‘Humid’ treatment. Reproducibility of results for the ‘Frozen’ treatment was 90 %.

**Conclusions:**

Despite the importance of specimen condition and the bisection step in determining sporozoite rates, little attention has been paid to them in the literature. Recommendations from this study are that: 1) care needs to be taken to reduce DNA degradation in the field; 2) mosquito abdomens be separated anterior to the junction of the thorax and abdomen; and 3) DNA sequencing of a subsample of positive results should be undertaken if possible.

## Background

*Anopheles* mosquitoes that harbor *Plasmodium* sporozoites in their salivary glands are potentially infectious to humans. Earlier stage oocysts that occur in the mosquito midgut may or may not develop into sporozoites, and oocyst rates are regarded as epidemiologically less informative as a measure of the potential of particular mosquito species to transmit malaria. Therefore, distinguishing between infected (oocysts only) and infective (with sporozoites) mosquitoes is important. Dissection is the traditional method for detecting oocysts in the midgut and sporozoites in the salivary glands. However, this method requires fresh specimens, experienced dissectors, and is generally unsuited to low endemicity areas where the processing of large numbers of mosquitoes is required. The development of the circumsporozoite protein antigen enzyme-linked immunosorbent assay (CS-ELISA) offered the possibility of high throughput, and high sensitivity and specificity [1], but this method can overestimate the true salivary gland infection rate and can give false-positives [1-5]. The use of molecular diagnostic tools is the most accurate and sensitive method for detecting malaria parasite species [6]. A single round method with PCR can detect as few as 10 sporozoites compared to 200–400 for CS antigen detection [7,8]. Rubio *et al*[9,10] described an even more sensitive semi-nested multiplex PCR, designed to identify the species of *Plasmodium* by using an initial genus-specific amplification followed by a secondary amplification that combines a universal *Plasmodium* primer and species-specific reverse primers. This technique can detect samples containing only 0.1 to 0.001 *Plasmodium* parasites per μL [9,10] or as few as three sporozoites [11] (or 0.06 pg DNA, assuming one genome equivalent of the parasite being 0.02 pg [12]). These protocols were designed to detect *Plasmodium* in human blood where high sensitivity is an advantage for detecting early stage infections. The most widely used molecular target for the detection of human *Plasmodium* infections, using a variety of PCR-based amplification methods, is the multicopy 18 S rRNA or small subunit nuclear ribosomal ribonucleic acid gene(s) (*ssrDNA*) [13-16]. Single nucleotide polymorphism (SNP) analysis and genomic mining are identifying new gene targets, with more copies that promise even more sensitive assays [6,17].

Application of these techniques for detecting infective stage mosquitoes and estimating sporozoite rates requires removal of the abdomen of the mosquito. For example, ELISAs detect CS proteins, which can be present in the developing oocysts and circulating in the haemolymph [18]. Mohanty *et al*[19] described a method for processing mosquitoes using two rounds of PCR, using separate spots on filter paper of the head-thoracic portion and the remaining abdominal parts. This arrangement allowed for the molecular identification of the vector, the bloodmeal host, and *Plasmodium* species. Head and thorax portions are typically subjected to PCR using standard *ssrDNA* markers, and it is assumed that all positive *Anopheles* mosquitoes have infective sporozoites [20,21].

Stoffels *et al*[22] showed that a single round PCR product coding for the 18 S rRNA gene hybridized to an oligonecleotide probe, was negative for head + thorax portions of *Anopheles gambiae* that contained *Plasmodium falciparum* oocysts (eight days post infection) but no sporozoites. Tsuzuki *et al*[23], in a study of *Plasmodium yoelii*, showed that PCR of the anterior portion of a mosquito thorax can result in a false-positive for mosquitoes that have recently fed on human blood infected with malaria (erythrocytic form). Despite the importance of the mosquito bisection step for excluding oocyst DNA in the abdomen, very little research has been directed to this method, and little detail about it is given in publications that report sporozoite rates.

The availability of highly sensitive PCR techniques and the lack of information regarding the effect of specimen handling are unexplored potential sources of error in studies that report sporozoite rates. In addition, sporozoite rates may be difficult to interpret particularly if mosquitoes are counted as positive when they have insufficient numbers of sporozoites (e.g. <10/salivary gland) to infect a human.

The aim of this study was to use standard semi-nested PCR of *ssrDNA* to determine the necessary conditions for removing mosquito abdomens in order to minimize false positives for *Plasmodium* sporozoites. The effect of different specimen storage conditions on rates of false negatives was also explored.

## Methods

### Mosquito infection

*Anopheles stephensi* that were either exposed or not exposed to potential infection were used. These mosquitoes were reared at the Walter Reed Army Institute of Research (WRAIR) insectary at 28 ± 1°C and 80 % r.h. Infections were obtained by membrane feeding on human blood mixed with a culture of *P. falciparum* strain NF54 at the Walter Reed Army Institute of Research. Mosquitoes were killed with CO_2_ , 6–7 days p.i. (post-infection) and examined for the presence of oocysts, or 14 days p.i. for sporozoites. The condition of the oocysts and salivary gland infections were examined in a subsample of the same cohort of infected mosquitoes using light microscopy. It should be noted that not all mosquitoes given infected blood will end up infected, even at the extended time period.

### Mosquito treatment

To simulate a variety of conditions that may be experienced by field-collected specimens, three mosquito killing/storage treatments were undertaken. These were: 1) mosquitoes were killed by freezing and kept frozen until bisection (i.e. the ‘Frozen’ treatment); 2) mosquitoes were killed by CO_2_, air dried for 24 h at room temperature, then kept frozen until bisection (i.e. ‘Dried’ treatment); and 3) a subsample of mosquitoes from treatment 2 was thawed then kept overnight at room temperature in a sealed plastic bag containing water-soaked paper tissues and then re-frozen until bisection (i.e. ‘Humid’ treatment). The Humid treatment was an attempt to simulate mosquitoes dying in an adult mosquito trap operating in an environment with high humidity. All preparations were conducted at 25°C in an air-conditioned laboratory. Mosquitoes from the control (uninfected) group were processed as in the Frozen treatment. Batches of mosquitoes with different levels of infection were used for the ‘Frozen’ treatment compared to the ‘Dried’ and ‘Humid’ treatments.

### Mosquito bisection

**Figure 1:**
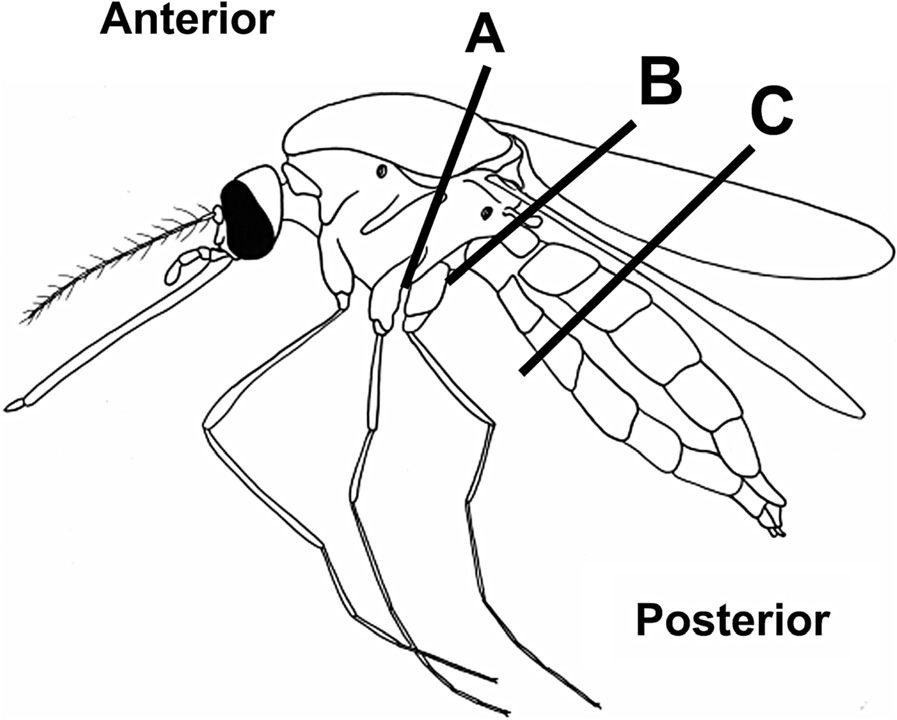
**Bisection positions**. Generalized mosquito showing bisection positions (A, B, C) and identity of portion (Anterior, Posterior) used to test, by PCR, for the presence of *Plasmodium falciparum* DNA.

Separation of mosquito abdomens is necessary to distinguish malaria infections of the midgut and salivary glands. To establish the best mosquito bisection technique and simulate some of the worst effects of possible human error, anterior and posterior portions of mosquitoes were obtained using different points of bisection (A, B, C, see Figure 1). In this case, location B is assumed to be the normal point of bisection.

Mosquito bisection was done under a dissecting microscope, and using fine forceps and a scalpel blade. Two sets of forceps and scalpel blades were alternated during each bisection session; with one set soaked in bleach (Sodium Hypochorite, 50 %) then rinsed in distilled water, while the other was in contact with the mosquito. Mosquitoes were bisected on a glass microscope slide that was first wiped clean with a tissue lightly soaked in bleach, then allowed to dry before the next dissection. Anterior and posterior portions of the mosquito were transferred by forceps to adjoining wells of a 96-well DNA block (AutoGen, Inc., Holliston, MA), with each well pre-filled with 300 μl of digestion buffers (M1 and M2, Autogen). Blocks were sealed with an aluminum foil lid and stored at −20°C until DNA extraction.

### DNA extraction

Mosquitoes were digested overnight at 56 ° C in a shaking incubator using the serene protease, proteinase K. Directly following digestion, DNA was extracted via phenol-chloroform extraction using the Autogen automated DNA extraction robot following the protocol for animal extractions. Following DNA extraction, samples were stored at −20 ° C until processing. The bottom right well was left as a negative control.

### Parasite detection

Specimens were tested for infection with malaria parasites using semi-nested multiplex PCR [1,9,10]. Amplification protocols were established for each primer pair using DNA from positive controls derived from *Plasmodium vivax* infected mosquitoes from the laboratory and from merozoite stage infected blood samples. Master mix components for *ssrRNA* amplification consisted of 1x buffer, 0.4 μM of each primer, 0.1 mM dNTP, 1.5 mM MgCl, 5 % DMSO, 1 unit of Biolase Taq, and 1 μl of DNA template. The total reaction volume was 20 μl. For the second round of PCR the same master mix was repeated. The cycling parameters were 94 ° C for 2 minutes followed by 35 cycles of 94 ° C for 30 seconds, 62 ° C for 30 seconds and 72 ° C for 1 minute. The final 72 ° C extension time was 7 minutes. The second PCR used the same cycling parameters with 40 cycles.

Primers PLF (5’-AGTGTGTATCAATCGAGTTTC- 3’) and UNR (5’-GACGGTATCTGATCGTCTTC-3’) were used to amplify a 783–821 basepair segment of the ssrRNA gene, common to all four human *Plasmodium* species, with sufficient variability to be suitable for species identification using oligonucleotide probes [24-26]. The second PCR used the same forward PLF primer and the *P. falciparum* specific reverse primer FAR (5’- AGTTCCCCTAGAATAGTTACA –‘3) which amplifies a 395 bp fragment.

PCR product (4 μl) was separated by electrophoresis on a 0.9 % agarose gel, stained with ethidium bromide, and bands were visualized by UV transillumination. A selection of positives were sequenced via Sanger sequencing using the species specific FAR and the universal PLF primers. Sequences were cleaned and aligned in Sequencher (Gene Codes Corp., Ann Arbor, MI) and run through a *Plasmodium Genomics Resource* BLAST [27] to confirm that amplification was indeed *P. falciparum*.

## Results

Results for the ‘Humid’, ‘Dry’, and ‘Frozen’ treatments are given in Tables 1, 2, and 3, respectively. The combined results are given in Table 4. These show occasional positives for sporozoites in specimens whose age post-infection (6–7 days p.i.) precluded this life stage. Over all treatments, a total of 40 positive samples were sequenced, of which 38 had a quality score higher than 90 %. All were a significant match to *P. falciparum*.

**Table 1:** PCR results for malaria parasites in mosquitoes from the ‘Humid’ treatment

**Infection stage (bisection position)**	**Numbers**				**Percent**			
	**Anterior**		**Posterior**		**Anterior**		**Posterior**	
	**+**	**-**	**+**	**-**	**+**	**-**	**+**	**-**
Control (B)	0	27	0	27	0	100	0	100
Oos only (C)	1*	25	0	26	3.8*	96.2	0	100
Oos only (B)	0	27	0	27	0	100	0	100
Oos only (A)	0	7	2	5	0	100	28.6	71.4
Spz + Oos (B)	2	25	0	27	7.4	92.6	0	100

* Corresponding abdomen was not positive.

Numbers of specimens and percentages positive or negative for *Plasmodium falciparum*, as determined by PCR, for the **‘**Humid’ treatment, for anterior and posterior portions of specimens of *Anopheles stephensi,* at different stages of infection (Oos only = oocysts only, 6–7 days p.i.; Spz + Oos = sporozoites and oocysts, 14 days p.i.), and different bisection locations (A, B, C, see Figure 1).

**Table 2:** PCR results for malaria parasites in mosquitoes from the ‘Dried’ treatment

**Infection stage (bisection position)**	**Numbers**				**Percent**			
	**Anterior**		**Posterior**		**Anterior**		**Posterior**	
	**+**	**-**	**+**	**-**	**+**	**-**	**+**	**-**
Control (B)	0	27	0	27	0	100	0	100
Oos only (C)	6*	16	14	8	27.3*	72.7	63.6	36.4
Oos only (B)	1**	25	4	22	3.8**	96.2	15.4	84.6
Oos only (A)	-	-	-	-	-	-	-	-
Spz + Oos (B)	40	12	-	-	76.9	23.1	-	-

* One corresponding abdomen was not positive; **Corresponding abdomen had unusual, larger bands.

Numbers of specimens and percentages positive or negative for *Plasmodium falciparum*, as determined by PCR, for the ‘Dried’ treatment, for anterior and posterior portions of specimens of *Anopheles stephensi,* at different stages of infection (Oos only = oocysts only, 6–7 days p.i.; Spz + Oos = sporozoites and oocysts, 14 days p.i.), and different bisection locations (A, B, C, see Figure 1).

**Table 3:** PCR results for malaria parasites in mosquitoes from the ‘Frozen’ treatment

**Infection stage (bisection position)**	**Numbers**				**Percent**			
	**Anterior**		**Posterior**		**Anterior**		**Posterior**	
	**+**	**-**	**+**	**-**	**+**	**-**	**+**	**-**
Control (B)	0	24	0	24	0	100	0	100
Oos only (C)	0	22	6	16	0	100	27.3	72.7
Oos only (B)	3*	41	5	39	6.8*	93.2	11.4	88.6
Oos only (A)	0	45	9	36	0	100	20.0	80.0
Spz + Oos (B)	23	30	28	25	43.4	56.6	52.8	47.2

*two corresponding abdomens were not positive or had faint bands. One of 3 was positive for abdomen in second replicate.

Numbers of specimens and percentages positive or negative for *Plasmodium falciparum*, as determined by PCR, for the ‘Frozen’ treatment, for anterior and posterior portions of specimens of *Anopheles stephensi,* at different stages of infection (Oos only = oocysts only, 6–7 days p.i.; Spz + Oos = sporozoites and oocysts, 14 days p.i.), and different bisection locations (A, B, C, see Figure 1).

**Table 4:** PCR results for malaria parasites in mosquitoes from combined treatments

**Infection stage (bisection position)**	**Numbers**				**Percent**			
	**Anterior**		**Posterior**		**Anterior**		**Posterior**	
	**+**	**-**	**+**	**-**	**+**	**-**	**+**	**-**
Control (B)	0	51	0	51	0	100	0	100
Oos only (C)	7*	63	20	50	10.0*	90.0	28.6	71.4
Oos only (B)	4*	93	9	88	4.1*	95.9	9.3	90.7
Oos only (A)	0	52	11	41	0	100	21.2	78.8
Spz + Oos (B)	65	67	28	52	49.2	50.8	35.0	65.0

*apparent false positives.

Numbers of specimens and percentages positive or negative for *Plasmodium falciparum*, as determined by PCR, across all treatments, for anterior and posterior portions of specimens of *Anopheles stephensi,* at different stages of infection (Oos only = oocysts only, 6–7 days p.i.; Spz + Oos = sporozoites and oocysts, 14 days p.i.), and different bisection locations (A, B, C, see Figure 1).

Multiple bands were noted for the ‘Frozen’ positive samples. Streaking, in keeping with DNA degradation, was noted for some ‘Humid’ samples. DNA degradation appears to have increased the rate of false negatives in the ‘Humid’ treatment, as the rate of positives for both mosquito portions is much lower than for the ‘Dried’ treatments (Tables 1 and 2). Bisection of dried specimens was sometimes difficult as fragmentation of the specimen around the point of bisection was possible. This may have contributed to the false positive rate, as fragments of posterior portions could have been accidently included with the anterior portion. However, even with non-dried specimens in the ‘Frozen’ treatment, false positives for anterior portions were noted (Table 3). Anterior and posterior portions of the same specimen could both be positive, negative, or mixed.

The PCR detection of parasites was repeated for specimens from the ‘Frozen’ treatment. Of 384 specimens in the ‘Frozen’ treatment; 60 were positive for parasites in both replicates (i.e. +/+), 287 were negative in both (−/−), 14 were positive in the first and negative in the second (+/−), and 23 were negative in the first and positive in the second (−/+). Thus, reproducibility, or concordance between replicates, was 90 % ((60 + 287)*100/384). Of the −/+ group, 78 % were posterior (abdominal) portions of the mosquito, whereas equal numbers of anterior and posterior specimens were present in the +/− group. A notable result of retesting was that one 6–7 days p.i. anterior portion fell in the +/− group.

## Discussion

For the mosquito stage of the malaria life cycle, the internal distribution of the parasite DNA is critical for inferring the infective status of the mosquito. Removal of the mosquito abdomen, where a potential contaminant, the oocyst DNA, is located is a crude but commonly accepted method for isolating the DNA of interest. Removal of abdomens has been used as an indirect method of determining sporozoite rates, under the assumption that only DNA from the infective stage of *Plasmodium* (i.e. the sporozoite) remains in infected samples. Stoffels *et al*[22] used a PCR method to determine that mosquitoes containing mature *P. falciparum* oocysts but not sporozoites gave no positive signal for the head + thorax. Although the numbers of mosquitoes that these authors tested was modest, and the stated detection limit of their method was 10 sporozoites per mosquito, this result suggested that only infective mosquitoes can be detected after removal of the abdomen.

Using the nested PCR method for *ssrDNA*, this study found apparent false positives for *P. falciparum* sporozoites in the head + thorax of infected mosquitoes that had not lived long enough to have developed this parasite stage. Bisecting mosquitoes anterior to the normal position resulted in zero false positives, suggesting that the bisection step is crucial. In addition, dissecting dried specimens was not optimal due to the brittle nature of the specimen, which sometimes makes a clean cut difficult to achieve. Dissection tools in this study were sterilized between operations but it was not clear how effective this step was. Sterilization is not normally mentioned in the methods section of papers reporting sporozoite rates but should be investigated.

In contrast to the false positive result, DNA deterioration under conditions that mimic those that could occur in mosquito traps under humid field conditions was found to increase false negatives, which would underestimate actual sporozoite rates.

The impact of false positives and false negatives would be expected to be greater in areas of low malaria endemicity, where the benefits of mass screening with a PCR detection method are greatest. False positives could cause an overestimation of the EIR, which can have important implications for vector incrimination, estimating malaria transmission, and the evaluation of vector control strategies [28]. In addition, if apparent sporozoite rates are spurious or are inflated by oocyst DNA, then efforts to determine the date of infection based on the minimum time to develop sporozoites [29] will be compromised.

Recently, two rounds of PCR, such as the nested PCR method for *ssrDNA* of Rubio *et al*[10] that is able to detect considerably less than one gametocyte per μl of blood, has been used for determining sporozoite rates [21]. Even more sensitive gene targets and methods are now being used [6,17,30]. These techniques have been developed to detect blood stage parasitemia, where higher sensitivity allows the early detection and treatment of infected humans. For mosquitoes, the question is how sensitive does a sporozoite detection method need to be? In particular, how does one balance the need to identify infective mosquitoes while reducing the chance of false positives through circulating sporozoites in the haemocoel or trace oocyst DNA that has contaminated the mosquito thorax during bisection? Mosquitoes with fewer than 100 sporozoites in the salivary glands infrequently ejected sporozoites under laboratory conditions [31,32]. Ito *et al*[33] reported that mean *P. vivax* sporozoite densities below 400 per salivary gland were insufficient to initiate infections in mice. If this is true for humans, a PCR method with a sensitivity of 10–100 sporozoites per mosquito appears to be more than sufficient to establish infectivity in most cases.

A limitation of the present study was that an indirect indicator of the presence of sporozoites based on knowledge of average parasite development rate was used, rather than a direct measure such as microscopic examination. As a result it is difficult to interpret PCR results in terms of their sensitivity and specificity. How do PCR results, let alone the results from retesting samples, relate to true parasite positivity? If only positive specimens from the first PCR (n = 74) were retested, 81.0 % would again test positive (n = 60), but this would miss 23.7 % (n = 23) of positives if all specimens were retested. Do specimens that only test positive in one out of two tests do so because of borderline quantities of DNA? If that is the case, then is may be better to skip retesting, err on the side of caution, and accept that some positives may be due to low (non-infective) numbers of sporozoites.

Rubio *et al*[10] speculated that false positives rarely (0.3 %) occur, although there was a chance of cross-contamination. Any PCR, especially consecutive rounds of amplification, may result in false-positives because of cross-contamination of samples. The present study undertook to minimize this risk by: the use of filtered pipette tips to move DNA, reagents and PCR template; having each 96-well plate with a positive and negative control; use of standardized robotic DNA extraction; use of pre-aliquoted and UV-irradiated water for PCR reactions; use of a multichannel pipettor to transfer DNA and template; the use of separate sterilized silicone and foil lids to cover 96-well plates between PCR1 and PCR2; centrifugation of plates prior to removal of lids to ensure that liquid was not on the lid or edges; and the re-testing of anomalous results. Positive and negative controls were always positive or negative by the nested PCR, however, a 1 in 10 chance of a different result was found upon retesting the ‘Fresh’ treatment.

Demas *et al*[6] reported that nonspecific bands can sometimes occur using the standard nested PCR method for *ssrDNA* with field collected blood samples. Bass *et al*[30] also found nonspecific bands that had been stored in ethanol and isopropanol, which likely increased the number of false positives. Rubio *et al*[10] also noted that discrepancies between microscopy and PCR only occurred in their field samples, and recommended drying blood samples rather than storing them frozen. Although, the present study tested mosquitoes rather than blood, it is interesting that ‘Frozen’ specimens also resulted in multiple bands in positive specimens.

Specimens under the ‘Humid’ treatment frequently gave smears on agarose gels. This treatment probably led to the denaturation of DNA, and possibly to microbial growth that may result in spurious bands. Lareaux *et al*[20], Arez *et al*[34] and others found that a major barrier to successful PCR, and a possible contributor to false negatives, are inhibitors still present after DNA extraction, especially from the exoskeleton of the mosquito head and thorax. Inhibitors and denaturation of DNA are expected to increase the rate of false negatives, whereas spurious bands may increase the rate of false positives.

Other possible reasons for false positives include: 1) error in specimen order, 2) contamination of 6–7 days p.i. batch with older (infected) specimens prior to dissection stage, and 3) insectary selection for faster parasite development. Specimens were not completely destroyed during DNA extraction and inspection confirmed that anterior and posterior portions were in the correct order, which suggests that the first possibility above is unlikely. Insectary protocols are in place to minimize the chance of mosquito batch contamination, although this is always a possibility. No information to test the third possibility is available.

A recommendation of this study is that extra care, such as by frequently emptying mosquito traps, needs to be taken to reduce DNA degradation in the field. Specimens should be bisected while they are fresh to ensure a clean separation of anterior and posterior portions, and preferably anterior to the junction of the thorax and abdomen. Storage in alcohol should make it easier to bisect specimens and may be more convenient in some situation. However, alcohol storage may reduce the usefulness of specimens for morphological identification, can present handling and safety issues in the field, presents problems if specimens are to be sent by airmail, and Bass *et al*[30] reports a negative effect of alcohol storage of specimens on PCR results. However, Hasan *et al*[1] found no difference in the amplification of the cytochrome B gene of *P. falciparum* and *P. vivax* when mosquitoes were stored in ethanol versus stored dry.

## Conclusions

Despite the importance of specimen condition and the bisection step in determining sporozoite rates, little attention has been reported about these in the literature. The present study found apparent false positives that would inflate the sporozoite rate are possible depending on where mosquito specimens are bisected. Recommendations from this study are that: extra care needs to be taken to reduce DNA degradation in the field; mosquito abdomens be separated anterior to the junction of the thorax and abdomen; and, a double check of anomalous results, and DNA sequencing of a subsample of positive results be undertaken if possible. As more sensitive PCR tests for *Plasmodium* are developed, the potential for false positives and inflated estimates of infective mosquitoes will increase. Finally, the sensitivity of the PCR method chosen for sporozoite detection should be no more than necessary to determine infectivity.

## Competing interests

The authors declare that they have no competing interests.

## Authors’ contributions

DF conceived, designed and coordinated the study, bisected the specimens, and drafted the manuscript, GH conducted the PCR and DNA sequencing, and helped draft the manuscript, JM coordinated delivery of specimens, and helped draft the manuscript, MD reared the specimens, and helped draft the manuscript, LR helped draft the manuscript, RW helped draft the manuscript, All authors read and approved the final manuscript.
